# Management and outcome across the spectrum of high‐risk patients with myocardial infarction according to the thrmobolysis in myocardial infarction (TIMI) risk‐score for secondary prevention

**DOI:** 10.1002/clc.23715

**Published:** 2021-09-01

**Authors:** Tzlil Grinberg, Tamir Bental, Yoav Hammer, Abid Assali, Hana Vaknin‐Assa, Maya Wiessman, Leor Perl, Ran Kornowski, Alon Eisen

**Affiliations:** ^1^ Department of Cardiology Rabin Medical Center Petah Tikva Israel; ^2^ Sackler Faculty of Medicine Tel Aviv University Tel Aviv Israel

**Keywords:** clinical outcomes, high‐risk populations, myocardial infarction, risk‐stratification, temporal trends

## Abstract

**Background:**

Patients with myocardial infarction (MI) are at increased risk for recurrent cardiovascular events, yet some patients, such as the elderly and those with prior comorbidities, are particularly at the highest risk. Whether these patients benefit from contemporary management is not fully elucidated.

**Methods:**

Included were consecutive patients with MI who underwent percutaneous coronary intervention (PCI) in a large tertiary medical center. Patients were stratified according to the thrombolysis in myocardial infarction (TIMI) risk score for secondary prevention (TRS2°P) to high (TRS2°P = 3), very high (TRS2°P = 4), or extremely high‐risk (TRS2°P = 5–9). Excluded were low and intermediate‐risk patients (TRS2°P < 3). Outcomes included 30‐day/1‐year major adverse cardiac events (MACE) and 1‐year mortality. Temporal trends were examined in the early (2004–2010) and late (2011–2016) time‐periods.

**Results:**

Among 2053 patients, 50% were high‐risk, 30% very high‐risk and 20% extremely high‐risk. Extremely high‐risk patients were older (age 74 ± 10 year) and had significant comorbidities (chronic kidney disease 68%, prior CABG 40%, heart failure 78%, peripheral artery disease 29%). Drug‐eluting stents and potent antiplatelets were more commonly used over time in all risk‐strata. Over time, 30‐day MACE rates have decreased, mainly attributed to the very high (11.3% to 5.1%, *p* = .006) and extremely high‐risk groups (15.9% to 8.0%, *p* = .016), but not the high‐risk group, with similar quantitative results for 1‐year MACE. The rates of 1‐year mortality remained unchanged in either group.

**Conclusion:**

Within a particularly high‐risk cohort of MI patients who underwent PCI, the implementation of guideline‐recommended therapies has improved over time, with the highest‐risk groups demonstrating the greatest benefit in outcomes.

## 1 INTRODUCTION

Patients who experience a myocardial infarction (MI) are at increased risk for recurrent cardiovascular events. Nevertheless, this risk is not similar in all patients, and it is determined by the patient's age, burden of coronary artery disease, and concomitant comorbidities. We previously demonstrated that post‐MI patients who were at a higher risk for recurrent cardiovascular events according to the thrombolysis in myocardial infarction (TIMI) risk score for secondary prevention (TRS2°P) derived the most benefit from the improved implementation of guideline‐directed care throughout a decade long.^1^, ^2^ This trend was observed despite the fact that these high‐risk patients were oftentimes undertreated compared with lower‐risk patients.

This inverse relationship between the estimated cardiovascular risk of patients and the delivery of guideline‐recommended therapies has long been recognized and referred to as the “risk‐treatment paradox.”^3^, ^4^, ^5^, ^6^ Other studies in recent years have indicated a similar trend of proportionally greater clinical benefit with guideline‐based therapies among those with higher baseline risk,^7^, ^8^, ^9^, ^10^, ^11^, ^12^, ^13^, ^14^, ^15^ which usually make the perceptually more complex‐to‐treat patients, including the elderly and patients with a tendency to bleeding.

Since prior studies have seldom included patients at the highest‐risk after an MI, it is not clear whether this trend applies to the very and extremely high‐risk patients—those who are not only the sickest and most comorbid but also with a far more pronounced risk for adverse events from treatment. In the FAST‐MI registry appropriate secondary prevention treatment and cardiac rehabilitation prescription at discharge were associated with larger relative risk reduction in clinical outcomes, particularly among highest‐risk patients (TRS2°P ≥ 5).^13^, ^14^ Similarly, the net clinical outcome with the antiplatelet vorapaxar was more pronounced in the high‐risk (TRS2°P ≥ 3) group of patients.^8^

We aimed to examine temporal trends over more than a decade in the treatment and outcome across the spectrum of high‐risk patients according to the TRS2°P in post‐MI patients. We hypothesized that this high‐risk group would demonstrate a graded benefit in clinical outcomes across the years.

## 2 METHODS

A single‐center retrospective cohort study including all consecutive patients identified from the percutaneous coronary intervention (PCI) registry of the Rabin Medical Center (RMC, a tertiary medical center in Israel), who had undergone PCI due to MI and were discharged alive during the years 2004–2016. The RMC's registry database entails all consecutive patients' demographic, clinical, and angiographic data, details of which, including data collection and protocol, were previously elaborated.^1^ Data collection was approved by the hospital ethics committee in compliance with the Declaration of Helsinki, with a waiver for the need of individual informed consent. The index date for inclusion in the study cohort was the date of the first PCI performed for the indication of acute MI during the study period. In the case of several interventions for a single patient during that time, only the first was included in the analysis. MI was defined according to the standard universal definitions available at the time of the index hospitalization.

The TRS2°P is a simple risk score incorporating nine clinical characteristics, each is assigned a single point in the total count. These characteristics include age ≥ 75, diabetes mellitus, hypertension, current smoking, peripheral artery disease, prior stroke, prior coronary artery bypass graft surgery (CABG), chronic heart failure, and chronic kidney disease (defined by modification of diet in renal disease [MDRD] as <60 ml/min). This score was devised relatively recently^8^ in order to predict a gradient of risk for major adverse cardiovascular events (MACE) at 3‐years post‐MI. It was later validated for secondary prevention in a number of studies^7^, ^9^, ^13^, ^14^, ^16^ demonstrating the ability to risk‐stratify patients for recurrent events and to distinguish a pattern of increasing benefit with optimal treatment.

This study included only patients with TRS2°P ≥ 3. Patients with TRS2°P < 3 were excluded, as well as patients who had missing data regarding one or more components of the TRS2°P.

Patients were then stratified for recurrent cardiovascular events by the TRS2°P to three groups: high‐risk (TRS2°P = 3), very high‐risk (TRS2°P = 4), and extremely high‐risk (TRS2°P ≥ 5). Temporal trends were examined in the early (2004–2010) and late (2011–2016) time‐periods, representing the advancement in the care of patients after an acute coronary syndrome brought about by the later era, when PCI, radial approach, potent antiplatelets, and high‐potency statins have become the standard of care. Clinical outcomes included 30‐day MACE, 1‐year MACE, and 1‐year mortality. MACE was a composite of death, MI, stroke, or unstable angina. Another analysis with MACE including two more outcomes—target vessel revascularization or CABG was also performed (Table S1).

### 2.1 Statistical analysis

All descriptive data presented, including baseline characteristics of patients, features and management of the index MI, and clinical outcomes, were stratified by the three TIMI groups. Continuous parameters were presented by the average and standard deviation. Ordinal and nominal parameters were presented by number (N) and percent (%). To estimate differences in continuous parameters one‐way ANOVA test was performed for normally distributed values or Kruskal‐Wallis test for abnormally distributed values. To estimate differences in categorical parameters, as well as to calculate temporal trends in treatment and outcomes, the Chi‐square test was used. When the assumptions required for the asymptotic method were not met, the Monte Carlo method was applied instead.

A logistic regression model was constructed to evaluate the probability of 30‐day MACE. 1‐year MACE and 1‐year mortality according to the TRS2°P and temporal trends were assessed by a Cox regression analysis and illustrated by Kaplan–Meier survival curves. A two‐sided alpha level of 0.05 was considered statistically significant. All statistical analyses were performed using IBM SPSS Statistics for Windows, Version 27.0 (Armonk, NY: IBM Corp, 2020).

## 3 RESULTS

Of 4921 post‐MI patients, 2053 patients (42%) with TRS2°P ≥ 3 were included in the current study. Of these, 50% (*n* = 1036) were classified as high‐risk, 30% (*n* = 602) as very high‐risk, and 20% (*n* = 415) as extremely high‐risk patients (Figure S1). Baseline characteristics of patients are presented in Table S2. Compared with the other groups, extremely high‐risk patients were older (mean age 74 ± 10 years) and had an exceedingly high burden of other comorbidities including hypertension (98%), diabetes mellitus (87%), dyslipidemia (30%), peripheral vascular disease (29%), prior MI (32%), chronic kidney disease (67%), prior CABG (40%), congestive heart failure (78%), and chronic obstructive pulmonary disease (18%), among others. They were taking more anticoagulants and had lower admission hemoglobin and hematocrit values.

With respect to the index MI, extremely high‐risk patients presented more often with non‐ST elevation MI and three‐vessel coronary disease on angiography compared with the other risk groups (Table 1). They had lower post‐procedural nadir hemoglobin/hematocrit levels, and required blood transfusions to a larger extent, although the absolute rates were still fairly low. At discharge from the hospital, they were less commonly prescribed potent P2Y12 inhibitors and received clopidogrel more often. Angiotensin‐converting enzyme inhibitors (ACEi) and angiotensin receptor blockers (ARBs) were also prescribed less often in extremely high‐risk patients. Statins were recommended equally and extensively across all risk groups while diuretics and oral anticoagulants (primarily vitamin K antagonists) far prevailed in the extremely high‐risk group.

**TABLE 1:** Characteristics of the index MI and medications at discharge

	TIMI risk score for secondary prevention			
*n* (%)	High risk TRS2°P = 3 (*n* = 1036)	Very high‐risk TRS2°P = 4 (*n* = 602)	Extremely high riskTRS2°P ≥ 5 (*n* = 415)	*p* value
STEMI on presentation	426 (41.1)	209 (34.7)	109 (26.3)	<.001
Three vessels on angiography	499 (48.3)	323 (53.7)	276 (66.7)	<.001
Balloon angioplasty	39 (3.8)	40 (6.6)	28 (6.7)	.076
Stenting type, BMS	553 (53.4)	326 (54.2)	214 (51.6)	.076
Stenting type, DES	442 (42.7)	235 (39)	171 (41.2)	.076
Required blood transfusions	2 (0.2)	8 (1.4)	6 (1.5)	.006
Minimal post‐PCI Hgb (mg/dl), mean ± *SD*	12.2 ± 2	11.3 ± 2.1	10.9 ± 2	<.001
Maximal % Hgb difference, mean ± *SD*	7.2 ± 8.7	8.7 ± 9.8	9.1 ± 10	<.001
Minimal post‐PCI HCT (%), mean ± *SD*	36.1 ± 5.7	33.6 ± 5.9	32.4 ± 5.8	<.001
Maximal % HCT difference, mean ± *SD*	7 ± 8.8	8.5 ± 9.7	9.1 ± 10	<.001
Medications at discharge				
Aspirin	1004 (96.9)	569 (94.5)	394 (94.9)	.04
Clopidogrel	794 (76.6)	484 (80.4)	359 (86.5)	<.001
Ticagrelor	134 (12.9)	68 (11.3)	38 (9.2)	.12
Prasugrel	94 (9.1)	28 (4.7)	10 (2.4)	<.001
Statins	1012 (97.7)	585 (97.2)	401 (96.6)	.5
ACEI/ARB	961 (92.8)	518 (86)	350 (84.3)	<.001
Beta blockers	940 (90.7)	537 (89.2)	369 (88.9)	.46
OAC	67 (6.5)	74 (12.3)	63 (15.2)	<.001

*Note*: Maximal %Hgb/HCT difference – percent difference between admission and minimal values.

Abbreviations: ACEI, angiotensin converting enzyme inhibitors; ARB, angiotensin receptor blockers; BMS, bare metal stent; DES, drug eluting stent; HCT, hematocrit; Hgb, hemoglobin; OAC, oral anticoagulation; PCI, percutaneous coronary intervention; *SD*, standard deviation; STEMI, ST elevation myocardial infarction.

The 30‐day MACE of extremely high‐risk patients was higher compared with very high and high‐risk patients (12.5%, 8.5%, and 4.1%, respectively, *p* < .001, Table 2). This was driven mostly by a higher proportion of cerebrovascular accidents and unstable angina (8.4%, 5.3%, and 2.2%, *p* < .001; 1.7%, 0.7%, and 0.4%, *p* = .029, respectively). As expected, outcomes in 1 year reflected the same graded risk; The rate of 1‐year MACE and each of its individual components (1‐year mortality included, Figure S2) was proportionally higher in extremely high‐risk patients compared with the very high and high‐risk groups (35.9%, 25.2%, and 14.7%, *p* < .001, respectively). It is also important to note that at 1‐year extremely high‐risk patients' mean hemoglobin levels were still lower compared with the other risk groups *(*Table 2 *)*.

**TABLE 2:** Clinical outcomes at 30 days and 1 year

	TIMI risk score for secondary prevention			
*n* (%)	High riskTRS2°P = 3 (*n* = 1036)	Very high risk TRS2°P = 4 (*n* = 602)	Extremely high riskTRS2°P ≥ 5 (*n* = 415)	*p* value
30‐day MACE	42 (4.1)	51 (8.5)	52 (12.5)	<.001
30‐day Mortality	11 (1.1)	9 (1.5)	10 (2.4)	.154
30‐day MI	6 (0.6)	8 (1.3)	2 (0.5)	.198
30‐day CVA	23 (2.2)	32 (5.3)	35 (8.4)	<.001
30‐day UAP	4 (0.4)	4 (0.7)	7 (1.7)	.03
1‐year MACE	152 (14.7)	152 (25.2)	149 (35.9)	<.001
1‐year mortality	69 (6.7)	73 (12.1)	71 (17.1)	<.001
1‐year MI	24 (2.3)	35 (5.8)	28 (6.7)	<.001
1‐year CVA	42 (4.1)	52 (8.6)	52 (12.5)	<.001
1‐year UAP	33 (3.2)	21 (3.5)	29 (7)	.003
1‐year Hgb* (mg/dl) mean ± *SD*	11.6 ± 2.2	10.6 ± 2.1	10.3 ± 2.1	<.001

*Note*: MACE was defined as death/MI/stroke/UAP. *40% missing values.

Abbreviations: CVA, cerebrovascular accident; Hgb, hemoglobin; MACE, major adverse cardiovascular events; MI, myocardial infarction; UAP, unstable angina.

Examining temporal trends in treatment throughout more than a decade, we compared an early (2004–2010) to a later time‐period (2011–2016). The implementation of guideline‐directed therapies has improved considerably during time (Table 3); among all risk levels, the use of ticagrelor and prasugrel rather than clopidogrel, and the use of drug‐eluting stents has significantly increased (from 26% to 62%, *p* < .001). The use of statins has increased as well, though modestly, as it was rather prevalent to begin with. In contrary, aspirin prescription has declined in the late time period for very high and extremely high‐risk patient groups. No difference in the prescription of beta blockers and ACEi/ARBs, which was fairly extensive across all risk strata, was observed between the early and late time‐periods.

**TABLE 3:** Temporal trends in guideline recommended therapies

	Entire cohort			High risk (TRS2°P = 3)			Very high risk (TRS2°P = 4)			Extremely high risk (TRS2°P ≥ 5)		
%	Early (*n* = 1172)	Late (*n* = 881)	*p*‐value	Early (*n* = 607)	Late (*n* = 429)	*p*‐value	Early (*n* = 326)	Late (*n* = 276)	*p*‐value	Early (*n* = 239)	Late (*n* = 176)	*p*‐value
Stenting type, BMS	69.2	32	<.001	70.8	28.7	<.001	70.2	35.1	<.001	63.6	35.2	<.001
Stenting type, DES	25.9	61.7	<.001	26	66.2	<.001	23	58	<.001	29.7	56.8	<.001
Statins	96.6	98.3	.018	97.5	97.9	.69	95.7	98.9	.018	95.4	98.3	.1
Aspirin	97.4	93.6	<.001	97.4	96.3	.3	97.2	91.3	.001	97.9	90.9	.001
Clopidogrel	97	56.8	<.001	98.7	45.5	<.001	94.5	63.8	<.001	96.2	73.3	<.001
Ticagrelor	0	27.2	<.001	0	31.2	<.001	0	24.6	<.001	0	21.6	<.001
Prasugrel	0	15	<.001	0	21.9	<.001	0	10.1	<.001	0	5.7	<.001
Beta blockers	88.8	91.4	.57	90	91.8	.3	88.3	90.2	.46	86.6	92	.08
ACEI/ARB	89.1	89.1	.97	92.3	93.5	.46	85.6	86.6	.7	85.8	82.4	.35

Abbreviations: ACEI, angiotensin converting enzyme inhibitors; ARB, angiotensin receptor blockers; BMS, bare metal stent; DES, drug eluting stent.

**FIGURE 1:**
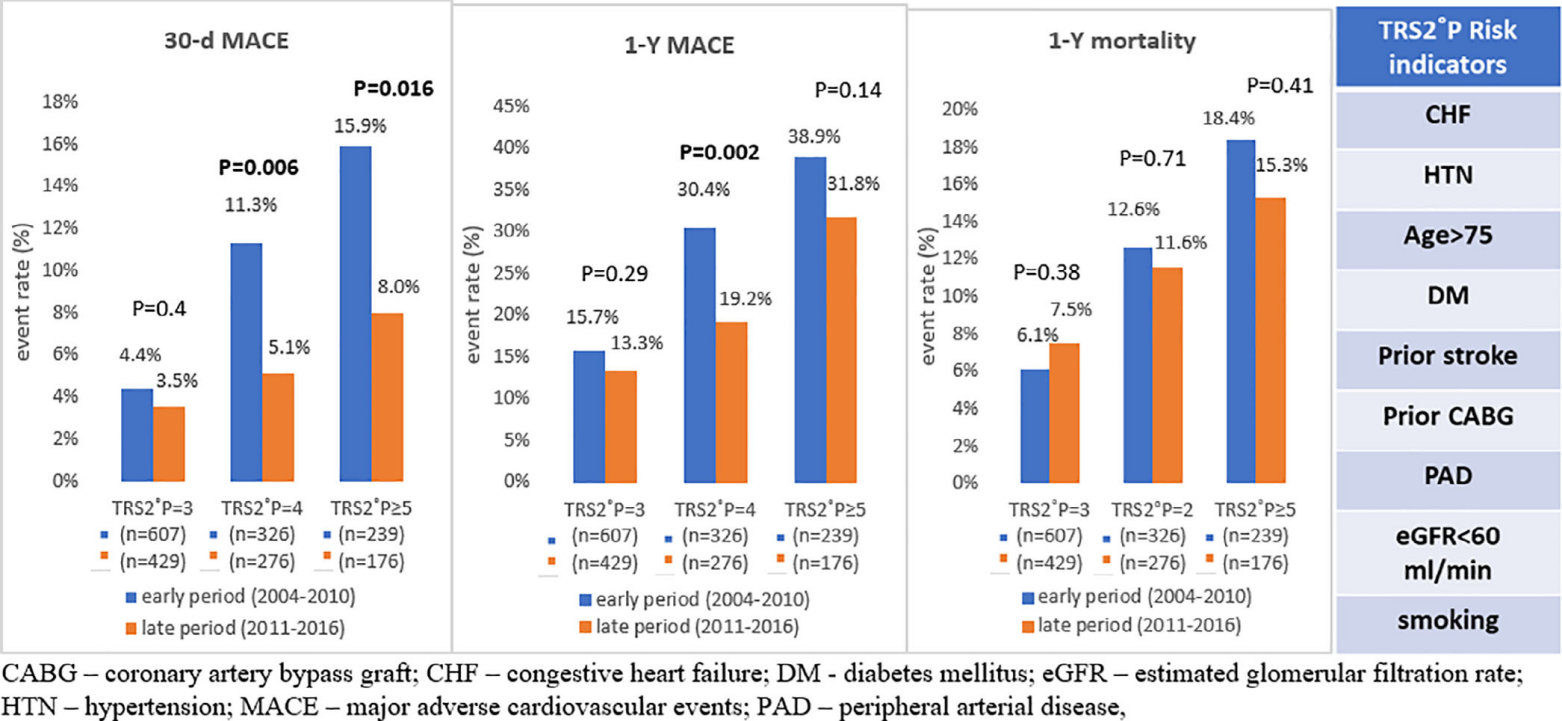
Temporal trends of 30‐day MACE, 1‐year MACE, and 1‐year mortality by the TIMI risk score for secondary prevention

**FIGURE 2:**
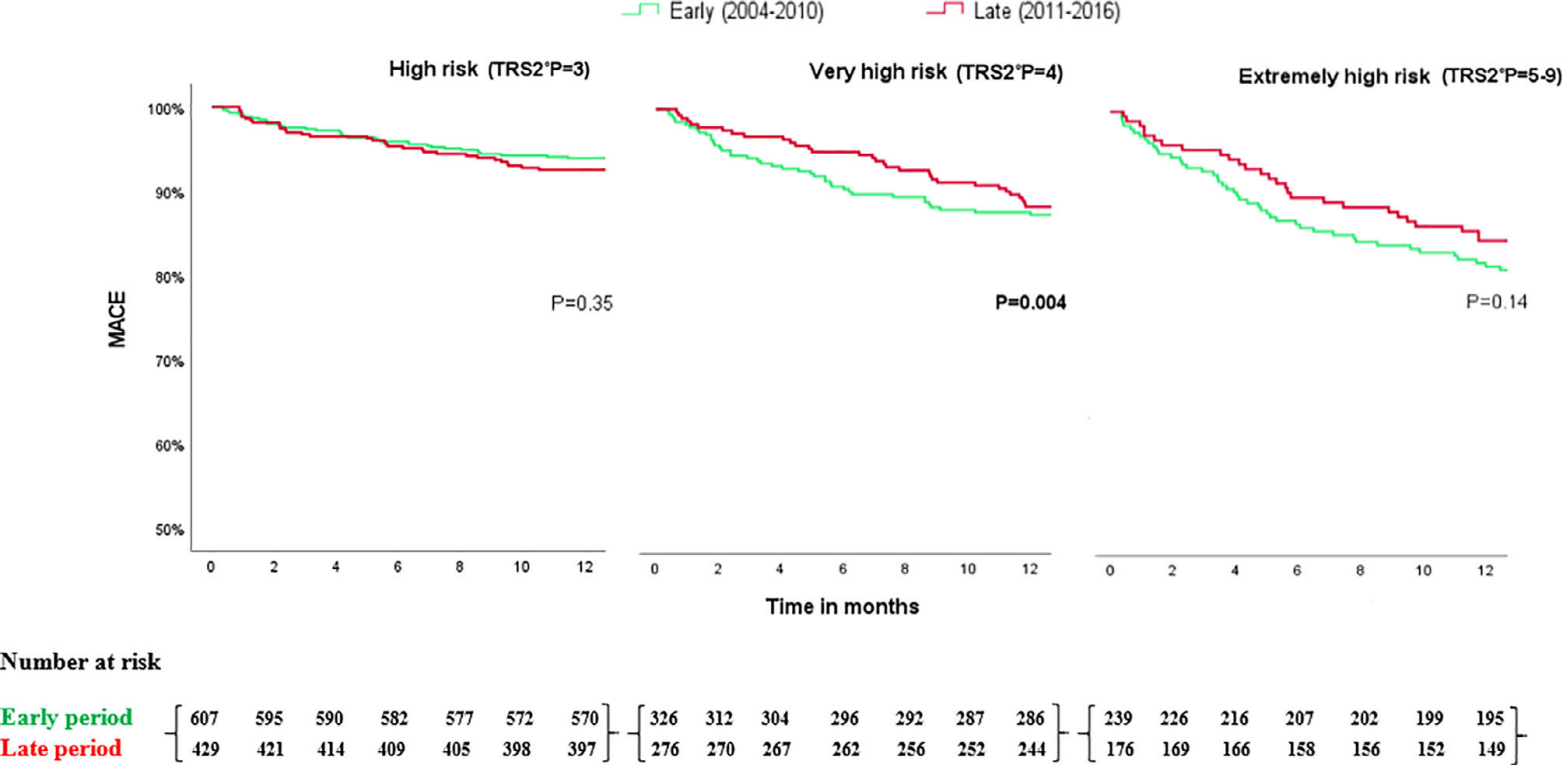
Kaplan–Meier curves for 1‐year MACE by time periods in high (TRS2°P = 3), very‐high (TRS2°P = 4), and extremely high‐risk patients (TRS2°P ≥ 5)

Examining temporal trends in clinical outcomes, the rate of 30‐day MACE has decreased in the entire cohort (Table S3). This reduction was mainly attributed to the extremely high‐risk (from 15.9% to 8.0%, *p* = .016) and the very high‐risk groups (from 11.3% to 5.1%, *p* = .006) but not to the high‐risk group, in which 30‐day MACE has not changed over time (from 4.4% to 3.5%, *p* = .44, Figure 1). The improved 30‐day MACE in the late period was driven largely by the decreased occurrence of cerebrovascular accidents. The rate of 1‐year MACE also decreased during time among the entire cohort, driven mainly by the very high‐risk group (from 30.4% to 19.2%, *p* = .002), but not among high or extremely high‐risk patients (Figure 2). The reduction in 1‐year MACE over time was driven by all of its individual components (MI, stroke, and unstable angina) except for the rate of 1‐year mortality, which did not change significantly from the early to the late period (Table S3). One‐year unstable angina rate particularly decreased over the late period among all risk groups.

In a logistic regression model adjusted for the TRS2°P and time period, the odds of 30‐day MACE decreased by 48% between the early and late time periods (odds ratio [OR] 0.52, 95% confidence interval [CI] 0.36–0.76, *p* = .001]. The extremely high‐risk group had a higher risk for 30‐day MACE regardless of the time period, but less so in the late than the early time period (OR 2.39, 95% CI 1.1–5.1 and OR 4.06, 95% CI 2.4–6.8, respectively), with no significant interaction between time period and TRS2°P.

In a Cox regression analysis, adjusted for the TRS2°P, 1‐year MACE decreased by 25% between the early and late time periods (Hazard ratio [HR] 0.75, 95% CI 0.62–0.91, *p* = .004) while 1‐year mortality has not changed (HR 0.97, 95% CI 0.74–1.27, P = 0.82).

## 4 DISCUSSION

In the present study, we demonstrated several findings; first, we substantiated the TRS2°P as a risk stratification tool that distinguishes a gradient of risk for MACE, not only in patients with prior MI, as was previously shown,^16^ but also when applied specifically to a high‐risk cohort. Second, we demonstrated that guideline‐recommended management, in particular the expanded use of drug‐eluting stents and potent antiplatelets, has improved considerably over time in all risk strata, albeit to a lesser extent with risk accrual. Discordantly, aspirin use decreased slightly (but significantly) over time to a greater extent with risk accrual, possibly as part of a single antiplatelet strategy in those at the extremes of bleeding risk. Third, we observed an improved 30‐day and 1‐year MACE over the late compared with the early period in the entire patient cohort. However, not all risk‐groups demonstrated the same benefit; with the high‐risk group, being the largest group, not displaying any benefit (despite a non‐statistically significant reduced MACE), while the very high‐risk and extremely high‐risk groups accounting for most of the benefit. Finally, none of the groups exhibited a reduction over time in 1‐year mortality.

In this high‐risk cohort of patients who were the subject of the current study, the utilization of drug‐eluting stents and potent antiplatelets has improved considerably, in keeping with findings from prior studies.^1^, ^2^, ^4^, ^6^ Furthermore, owing to the study design, all included patients had undergone PCI, and while this was an inclusion criteria and not a treatment aspect to be compared between time periods, it is still noteworthy as revascularization is often underutilized in elderly and comorbid populations^17^, ^18^ (although the benefit appears to be maintained at older age^3^, ^19^) and as it probably did have a profound impact on these patients' outcomes.

We chose to focus on patients who are at the highest risk for recurrent cardiovascular events—A population who, due to the complexity of their comorbidities, poses a therapeutic challenge; they are uncommonly enrolled in randomized clinical trials,^20^ they are oftentimes managed conservatively rather than invasively with angiography, and are usually infrequently treated with guideline‐directed medical therapy. They ultimately experience higher rates of morbidity and mortality, far exceeding the previously reported 10‐year atherosclerotic cardiovascular disease (ASCVD) risk^21^, ^22^ and the 10‐year risk of fatal cardiovascular outcomes^23^ attributed to very high‐risk and extremely high‐risk patients. Still, we found no studies specifically addressing the optimal management of these particularly high‐risk patients, and most of the data were driven from subgroup analysis of other populations.^8^, ^24^

The extremely high‐risk patients in this study had significant co‐morbidities—the majority had hypertension, diabetes mellitus, heart failure, and chronic kidney disease. In addition, about a third had prior CABG and prior MI or stroke. Indeed, the excessive cardiovascular risk was mirrored in their associated significant 1‐year MACE rate of 35.9%, that consisted of the traditional three‐point MACE (nonfatal MI, nonfatal stroke, and mortality, the latter amounting to over 17%) along with the rate of unstable angina.

With respect to clinical outcomes, we demonstrated that the rate of 30‐day MACE has improved over time‐periods in very high‐risk and extremely high‐risk patients while it did not change significantly for high‐risk patients. This finding is consistent with prior studies of lower‐risk populations.^1^, ^2^ Likewise, the rate of 1‐year MACE decreased for very high‐risk patients while it did not change in high‐risk patients. Yet, extremely high‐risk patients did not show a significant benefit with regards to 1‐year MACE over time. This could partly be explained by their extreme risk for cardiovascular events—perhaps the burden of these patients' comorbidities was too high that in the long run it overwhelmed any benefit that the enhanced implementation of guideline‐directed treatment might have had on their general prognosis (mortality included). Attesting to this hypothesis is the fact that the short‐term outcome of 30‐day MACE did decrease in the late period among extremely high‐risk patients. In addition, the relatively small number of patients in this group may have had a bearing on the outcome considering the positive trend in the rate of 1‐year MACE (from 38.9% in the early period to 31.8% in the late period) and the decreased 1‐year unstable angina rate (from 10.5% to 2.3%, *p* = .001).

In view of the above, it is difficult to draw firm conclusions as to the presence of a graded benefit in clinical outcomes with higher patient risk within the high‐risk patient group specifically. However, for the general cohort included in this study, clinical outcomes have changed for the better throughout the two‐time periods, despite these patients' multitude of cardiovascular conditions and risk factors.

This study is based on a large registry of consecutive patients from a tertiary medical center. The data are complete and well documented. Nonetheless, our study has several limitations, including those inherent to a retrospective single‐center study. First, the data on medication use pertained only to the medications at discharge as data with respect to patient adherence to treatment were unavailable. Therefore, we cannot vouch for medication adherence post‐discharge date, though many of these patients continued follow‐up in the hospital clinics. Second, we had no available information regarding the number, location, and specific types of stents deployed, nor did we pertain data concerning periprocedural bleeding complications. Nevertheless, we did obtain data regarding blood transfusions received and hemoglobin levels at several time points during hospitalization and at 1‐year follow‐up. In addition, we lacked data with respect to rehabilitation referral, an important part of guideline‐directed management. Finally, our study cannot infer a causal relationship between the improved treatment and outcome.

## 5 CONCLUSION

Within a cohort of patients with MI at high, very high, and extremely high‐risk for recurrent cardiovascular events, the implementation of guideline‐recommended therapies has improved over a decade long, with the higher‐risk groups demonstrating the greatest benefit in cardiovascular clinical outcomes.

## CONFLICT OF INTEREST

The authors declare that there is no conflict of interest.

## AUTHORS' CONTRIBUTIONS

Alon Eisen and Ran Kornowski contributed to the conception of the work. Alon Eisen, Yoav Hammer, and Tzlil Grinberg contributed to its design. Tamir Bental, Abid Assali, Hana Vaknin‐Assa, Maya [Wiessman, and Leor Perl contributed to the acquisition of data for the work. Tzlil Grinberg drafted the manuscript and, together with Alon Eisen, contributed to the analysis and interpretation of data. Alon Eisen critically revised the manuscript, along with Ran Kornowski and Maya Wiessman. All the above gave final approval and agreed to be accountable for all aspects of the work ensuring integrity and accuracy.

## Supporting information


**Figure S1** Patient distribution by the TIMI risk score for secondary prevention.
**Figure S2.** Kaplan–Meier curves for 1‐year mortality by the TIMI risk score for secondary prevention.
**Table S1.** Clinical outcomes with MACE defined as death/MI/stroke/UAP/revascularization (TVR/CABG)
**Table S2:** Baseline characteristics by TRS2°P
**Table S3:** Temporal trends in clinical outcomes

## Data Availability

The data that support the findings of this study are available from the corresponding author upon reasonable request.
