# Supplementary material for: Management and outcome across the spectrum of high‐risk patients with myocardial infarction according to the thrmobolysis in myocardial infarction (TIMI) risk‐score for secondary prevention
Source: Clin Cardiol. 2021 Sep 1;44(11):1535–42. doi: 10.1002/clc.23715 (PMC8571543; doi:10.1002/clc.23715)

**Supplemental Materials**

|  | | | | TIMI risk score for secondary prevention | | | | | | | |  | | |
| --- | --- | --- | --- | --- | --- | --- | --- | --- | --- | --- | --- | --- | --- | --- |
| n (%) | | | | High risk  (TRS2˚P=3) (n =1036) | | | Very high risk  (TRS2˚P=4) (n=602) | | | Extremely high risk (TRS2˚P≥5) (n=415) | | P value | | |
|  | |  | | | | | | | | | | | |  |
| 30-day MACE | | | | 53 (5.1) | | | 59 (9.8) | | | 54 (13) | | **<0.001** | |  |
| 1-year MACE | | | | 208 (20.1) | | | 180 (29.9) | | | 167 (40.2) | | **<0.001** | |  |
| 1-year revascularization | | | | 84 (8.1) | | | 59 (9.8) | | | 49 (11.8) | | 0.08 | |  |
|  |  | | | | |  | | |  | | | |  |  |
| n (%) | Early (n=607) | | Late (n=429) | | P-value | Early (n=326) | Late (n=276) | P-value | Early (n=239) | | Late (n=176) | P-value |  |  |
| 30-day  MACE | 35 (5.8) | | 18 (4.2) | | 0.26 | 43 (13.2) | 16 (5.8) | **0.002** | 38 (15.9) | | 16 (9.1) | **0.04** |  |  |
| 1-year  MACE | 129 (21.3) | | 79 (18.4) | | 0.26 | 120 (36.8) | 60 (21.7) | **<0.001** | 98 (41) | | 69 (39.2) | 0.71 |  |  |
| 1-year revascular-ization | 58 (9.6) | | 26 (6.1) | | 0.04 | 45 (13.8) | 14 (5.1) | <0.001 | 28 (11.7) | | 21 (11.9) | 0.95 |  |  |
|  |  | |  | |  |  |  |  |  | |  |  |  |  |

**Table S1.** Clinical outcomes with MACE defined as death/MI/stroke/UAP/revascularization (TVR/CABG)

BMS – bare metal stent; CABG – coronary artery bypass grafting; DES – drug eluting stent; MACE – major adverse cardiovascular events; STEMI - ST elevation myocardial infarction; TVR – target vessel revascularization

**Table S2:** Baseline characteristics by TRS2°P

| TIMI risk score for secondary prevention | | |  |
| --- | --- | --- | --- |
| extremely high risk **TRS2˚P≥5** (n=415) | Very high risk **TRS2˚P=4** (n=602) | High risk **TRS2˚P=3**  (n =1036) | n (%) |
| 74.1±10.4 | 71.9±12 | 60.3±12.4 | Age (years), mean±SD |
| 284 (68.4) | 404 (67.1) | 756 (73) | Male |
| 408 (98.3) | 583 (96.8) | 943 (91) | Hypertension |
| 360 (86.7) | 414 (68.8) | 618 (59.7) | Diabetes mellitus |
| 127 (30.6) | 142 (23.6) | 273 (26.4) | Dyslipidemia |
| 74 (17.8) | 95 (15.8) | 98 (9.5) | COPD |
| 166 (40) | 236 (39.2) | 519 (50.1) | Smoking History |
| 121 (29.2) | 62 (10.3) | 46 (4.4) | PVD |
| 280 (67.5) | 278 (46.2) | 196 (18.9) | CKD |
| 55.6±28.2 | 67.2±28.9 | 83.9±27.9 | eGFR (ml/min), mean±SD |
| 132 (31.8) | 139 (23.1) | 132 (12.7) | Prior MI |
| 166 (40) | 102 (16.9) | 86 (8.3) | Prior CABG |
| 325 (78.3) | 345 (57.3) | 334 (32.2) | Prior CHF |
| 60 (14.5) | 78 (13) | 105 (10.1) | Prior AF |
| 136 (32.8) | 92 (15.3) | 65 (6.3) | Prior Stroke |
| 63 (15.2) | 55 (9.1) | 36 (3.5) | Prior Anticoagulation |
| 12±1.9 | 12.4±1.9 | 13.2±1.8 | Hgb at admission (mg/dL), mean± SD |
| 36±5.6 | 37±5.5 | 39±5.2 | HCT at admission (%), mean± SD |

P < 0.05 for each variable. AF – atrial fibrillation; CABG – coronary artery bypass graft; CHF – congestive heart failure; CKD – chronic kidney disease; COPD – chronic obstructive pulmonary disease; eGFR: estimated glomerular filtration rate; HCT – hematocrit; Hgb – hemoglobin; PVD – peripheral vascular disease; SD – standard deviation. CKD was defined as eGFR< 60 ml/min by MDRD formula.

**Table S3:** Temporal trends in clinical outcomes

|  | Entire Cohort | | | High risk  (TRS2˚P=3) | | | Very high risk  (TRS2˚P=4) | | | Extremely high risk (TRS2˚P≥5) | | | |
| --- | --- | --- | --- | --- | --- | --- | --- | --- | --- | --- | --- | --- | --- |
| n (%) | Early (n=1172) | Late (n=881) | P-value | Early (n=607) | Late (n=429) | P-value | Early (n=326) | Late (n=276) | P-value | Early (n=239) | Late (n=176) | P-value |  |
| 30-day  MACE | 102 (8.7) | 43 (4.9) | **0.001** | 27 (4.4) | 15 (3.5) | 0.44 | 37 (11.3) | 14 (5.1) | **0.006** | 38 (15.9) | 14 (8) | **0.016** |  |
| 30-day Mortality | 19 (1.6) | 11 (1.2) | 0.48 | 6 (1) | 5 (1.2) | 0.78 | 6 (1.8) | 3 (1.1) | 0.45 | 7 (2.9) | 3 (1.7) | 0.42 |  |
| 30-day MI | 12 (1) | 4 (0.5) | 0.15 | 6 (1) | 0 (0) | 0.039 | 6 (1.8) | 2 (0.7) | 0.23 | 0 (0) | 2 (1.1) | 0.1 |  |
| 30-day CVA | 61 (5.2) | 29 (3.3) | **0.036** | 12 (2) | 11 (2.6) | 0.52 | 23 (7.1) | 9 (3.3) | **0.039** | 26 (10.9) | 9 (5.1) | **0.037** |  |
| 30-day UAP | 12 (1) | 3 (0.3) | 0.07 | 4 (0.7) | 0 (0) | 0.09 | 2 (0.6) | 2 (0.7) | 0.86 | 6 (2.5) | 1 (0.6) | 0.13 |  |
| 1-year MACE | 287 (24.5) | 166 (18.8) | **0.002** | 95 (15.7) | 57 (13.3) | 0.29 | 99 (30.4) | 53 (19.2) | **0.002** | 93 (38.9) | 56 (31.8) | 0.14 |  |
| 1-year  mortality | 122 (10.4) | 91 (10.3) | 0.95 | 37 (6.1) | 32 (7.5) | 0.38 | 41 (12.6) | 32 (11.6) | 0.71 | 44 (18.4) | 27 (15.3) | 0.41 |  |
| 1-year  MI  1-year  CVA | 60 (5.1)  92 (7.8) | 27 (3.1)  54 (6.1) | 0.022  0.13 | 17 (2.8)  24 (4) | 7 (1.6)  18 (4.2) | 0.22  0.85 | 26 (8)  35 (10.7) | 9 (3.3)  17 (6.2) | **0.014**  **0.046** | 17 (7.1)  33 (13.8) | 11 (6.2)  19 (10.8) | 0.73  0.36 |  |
| 1-year  UAP | 70 (6) | 13 (1.5) | **<0.001** | 29 (4.8) | 4 (0.9) | **0.001** | 16 (4.9) | 5 (1.8) | **0.039** | 25 (10.5) | 4 (2.3) | **0.001** |  |

CVA – cerebrovascular accident; MACE – major adverse cardiovascular events; MI – myocardial infarction; UAP – unstable angina.

**Figure S1:** Patient distribution by the TIMI risk score for secondary prevention.


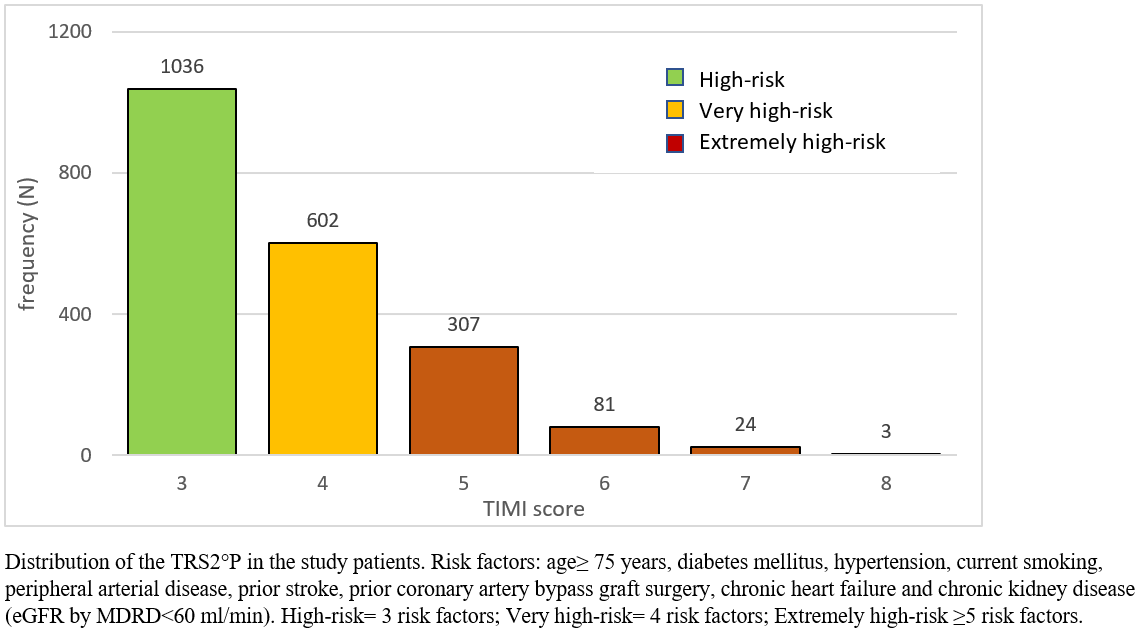


**Figure S2.** Kaplan-Meier curves for 1-year mortality by the TIMI risk score for secondary prevention.


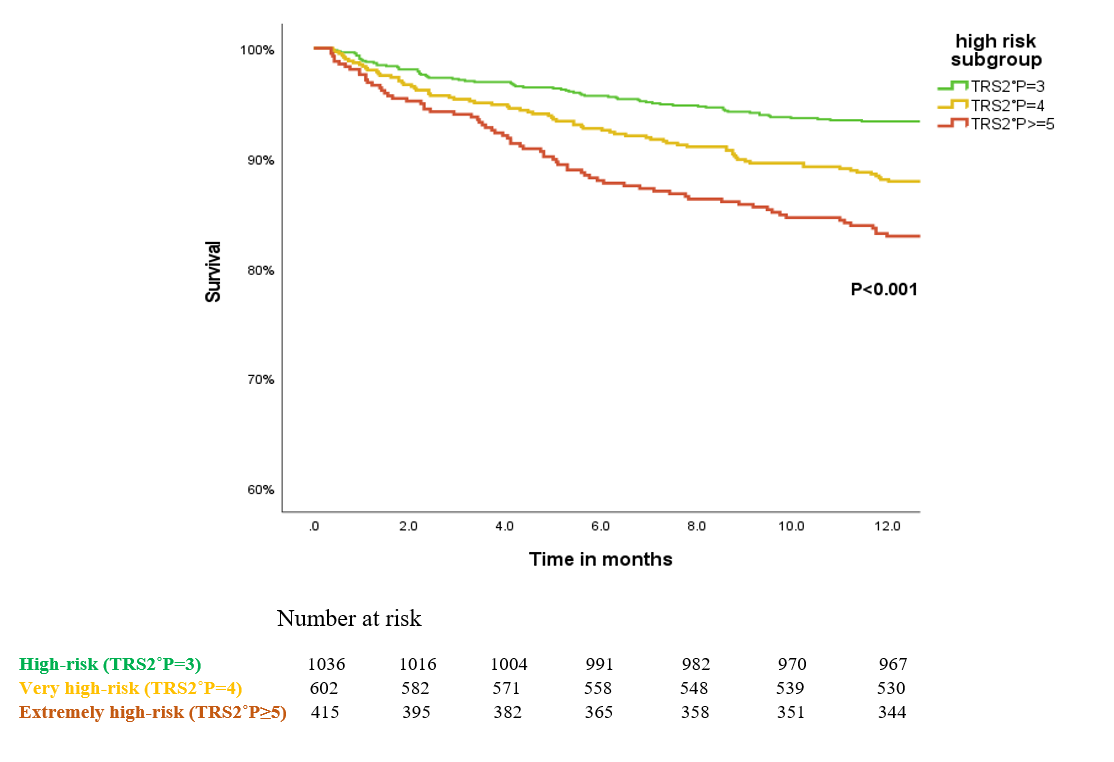

Supplement: Supplementary file 1 — Figure S1 Patient distribution by the TIMI risk score for secondary prevention. Figure S2. Kaplan–Meier curves for 1‐year mortality by the TIMI risk score for secondary prevention. Table S1. Clinical outcomes with MACE defined as death/MI/stroke/UAP/revascularization (TVR/CABG) Table S2: Baseline characteristics by TRS2°P Table S3: Temporal trends in clinical outcomes [file CLC-44-1535-s001.docx]
